# Risk of developing active tuberculosis following tuberculosis screening and preventive therapy for Tibetan refugee children and adolescents in India: An impact assessment

**DOI:** 10.1371/journal.pmed.1003502

**Published:** 2021-01-19

**Authors:** Kunchok Dorjee, Sonam Topgyal, Tenzin Tsewang, Tenzin Tsundue, Tenzin Namdon, Elizabeth Bonomo, Caroline Kensler, Dekyi Lhadon, Tsering Choetso, Tenzin Nangsel, Tsering Dolkar, Thupten Tsekyi, Chungdak Dorjee, Dawa Phunkyi, Tsetan D. Sadutshang, Zorba Paster, Richard E. Chaisson

**Affiliations:** 1 Center for TB Research, Division of Infectious Diseases, School of Medicine, Johns Hopkins University, Baltimore, Maryland, United States of America; 2 Division of Tuberculosis, Delek Hospital, Department of Health, Central Tibetan Administration, Dharamsala, India; 3 Tibetan Children’s Village School, Dharamsala, India; 4 Department of Family Medicine, University of Wisconsin, Madison, Wisconsin, United States of America; PLOS Medicine Editorial Board, UNITED STATES

## Abstract

**Background:**

Tuberculosis (TB) rates among Tibetan refugee children and adolescents attending boarding schools in India are extremely high. We undertook a comprehensive case finding and TB preventive treatment (TPT) program in 7 schools in the Zero TB Kids project. We aimed to measure the TB infection and disease burden and investigate the risk of TB disease in children and adults who did and did not receive TPT in the schools.

**Methods and findings:**

A mobile team annually screened children and staff for TB at the 7 boarding schools in Himachal Pradesh, India, using symptom criteria, radiography, molecular diagnostics, and tuberculin skin tests. TB infection (TBI) was treated with short-course regimens of isoniazid and rifampin or rifampin. TB disease was treated according to Tibetan and Indian guidelines. Between April 2017 and December 2019, 6,582 schoolchildren (median age 14 [IQR 11–16] years) and 807 staff (median age 40 [IQR 33–48] years) were enrolled. Fifty-one percent of the students and 58% of the staff were females. Over 13,161 person-years of follow-up in schoolchildren (median follow-up 2.3 years) and 1,800 person-years of follow-up in staff (median follow-up 2.5 years), 69 TB episodes occurred in schoolchildren and 4 TB episodes occurred in staff, yielding annual incidence rates of 524/100,000 (95% CI 414–663/100,000) person-years and 256/100,000 (95% CI 96–683/100,000) person-years, respectively. Of 1,412 schoolchildren diagnosed with TBI, 1,192 received TPT. Schoolchildren who received TPT had 79% lower risk of TB disease (adjusted hazard ratio [aHR] 0.21; 95% CI 0.07–0.69; *p* = 0.010) compared to non-recipients, the primary study outcome. Protection was greater in recent contacts (aHR 0.07; 95% CI 0.01–0.42; *p* = 0.004), the secondary study outcome. The prevalence of recent contacts was 28% (1,843/6,582). Two different TPT regimens were used (3HR and 4R), and both were apparently effective. No staff receiving TPT developed TB. Overall, between 2017 and 2019, TB disease incidence decreased by 87%, from 837/100,000 (95% CI 604–1,129/100,000) person-years to 110/100,000 (95% CI 36–255/100,000) person-years (*p <* 0.001), and TBI prevalence decreased by 42% from 19% (95% CI 18%–20%) to 11% (95% CI 10%–12%) (*p <* 0.001). A limitation of our study is that TB incidence could be influenced by secular trends during the study period.

**Conclusions:**

In this study, following implementation of a school-wide TB screening and preventive treatment program, we observed a significant reduction in the burden of TB disease and TBI in children and adolescents. The benefit of TPT was particularly marked for recent TB contacts. This initiative may serve as a model for TB detection and prevention in children and adolescents in other communities affected by TB.

## Abstract 2

Kunchok Dorjee and colleagues investigate infection and disease burden following mass tuberculosis preventive treatment for Tibetan refugee children at schools in India.

## Author summary

**Why was this study done?:**

- Tuberculosis (TB) is the world’s leading infectious disease killer, with estimated 10 million cases and 1.5 million deaths annually. Annually, 1 million children develop TB and 233,000 children die of the disease.
- Of the eligible children, only around 23% receive TB preventive treatment (TPT) globally.
- Knowledge of TB disease progression and the effectiveness of rifampin-based short-course TPT regimens in children and adolescents is lacking or limited globally.
- Operational experience on TPT implementation in high-burden, resource-poor settings is crucial for national programs.
- Tibetan refugee children and adolescents in India have extremely high rates of TB disease (853/100,000 person-years) and infection (19%).

**What did the researchers do and find?:**

- A comprehensive active case finding and TPT program was implemented for children, adolescents, and staff members of Tibetan boarding schools in India.
- Children and adolescents who received TPT with 3 months of daily isoniazid and rifampin (3HR) or 4 months of daily rifampin (4R) had 79% lower risk of TB disease; the protection was greater (93%) in participants with recent exposure to TB.
- The schools saw an 87% decline in TB disease incidence and a 42% decline in infection prevalence at the end of 2019.
- Both the 3HR and 4R regimens were apparently effective; the latter had fewer side effects.

**What do these findings mean?:**

- Children and adolescents constitute vulnerable populations with high TB disease burden requiring greater global attention.
- Recently exposed individuals can be targeted for preventive treatment in the global scale-up of TPT implementation.
- In this study, large-scale rifampin-based TPT was operationally feasible in resource-limited settings; well accepted by children, adolescents, and adults; and valuable in preventing TB.
- A multipronged approach anchored by TPT effectively reduced TB burden for a vulnerable refugee population, presenting an adaptable model for other communities.

## Introduction

It is estimated that every year 1.5 million children and adolescents 0–19 years old develop tuberculosis (TB) disease, and 7.5 million children and young adolescents (0–14 years) develop TB infection (TBI) [1–4]. Approximately 233,000 children and young adolescents die of TB annually [1,5]. Despite this disease burden, implementation of TB diagnostic and preventive services in children and adolescents remains suboptimal. Tibetan refugee children and adolescents in India constitute a vulnerable population having several demographic, socioeconomic, biomedical, and political risk factors for TB [6–9]. A drug-resistant TB survey carried out in the Tibetan population in India in 2010–2011 showed a high prevalence of multidrug-resistant TB (MDR-TB)—14.5% among new TB cases and 31.4% among previously treated cases [8]. In 2011–2013, during a community-wide active case finding campaign in India, we detected a high prevalence of TB disease in the Tibetan schools (394/100,000 schoolchildren) [6]. Driven by an urgent need to reduce the TB burden for this vulnerable population, and in response to the global campaign to end TB, we initiated a comprehensive TB screening and treatment program—Zero TB Kids (ZTBK)—for Tibetan children in India in 2017 [9]. During initial screening in 2017–2018, we detected a high prevalence of TB disease (916/100,000 schoolchildren) and TBI (19%) in children at Tibetan boarding schools in Himachal Pradesh, where children reside in congregate settings [9]. As a core component of this population-level program across schools, TB preventive treatment (TPT) was implemented using short-course regimens, with a high treatment completion rate (95%). ZTBK then continued with annual screening, treatment, and preventive treatment for TB. We present here the results and impact of 3 years of TB screening and TPT implementation under the program.

## Methods

### Project setting and population

The project is based at the Delek Hospital in Dharamsala, India, and aims to eliminate TB in Tibetan children. Tibetan children in India attend a network of boarding schools established by the Central Tibetan Administration (CTA) with support from the Government of India. These schools are home to children from prekindergarten to grade 12, many of whom are from poor families living in settlements across India and Nepal. In addition to modern education, the schools provide cultural and spiritual instruction to the children. Children are cared for by home mothers in dormitories and hostels within the school campus that are segregated by grade level and age. Staff members reside in residential quarters. All children and staff were eligible for screening and treatment under ZTBK. A mobile team comprised of medical officers and field nurses traveled to the schools and performed screening for active and latent TB and provided TPT as indicated. Children diagnosed with active TB were referred to the nearest Department of Health treatment center for TB treatment and remained in respiratory isolation until no longer deemed potentially infectious. Children diagnosed with latent TBI were provided TPT at the school. School nurses supervised active TB treatment, and home mothers supervised TPT for the children. All TB services were provided free of cost.

### Ethics statement

The study was ruled exempt as a public health initiative by the Johns Hopkins Medicine Institutional Review Board and approved by the CTA Department of Health, the CTA Department of Education, and each school administration. Oral permission was sought and received from parents or guardians by program staff for all children before providing TPT. Identifying information of the participants is maintained by the Department of Health, and only anonymized data were provided to the investigators. The initiation of the ZTBK collaboration has been previously described [9].

### Screening for TBI and disease

Between April 2017 and December 2019, 7 Tibetan boarding schools in Himachal Pradesh had periodic screening for TBI and TB disease. The schoolchildren and staff constitute an open cohort, with participants entering and exiting through new admissions in the schools and matriculation to college, respectively. Three waves of screening and treatment for latent TBI have been completed. In the starting year, 2017, all children and staff from the 7 boarding schools were screened for TBI, and children with TBI were offered TPT. In 2018, children newly admitted to the schools were screened for TBI and offered TPT. In 2019, all children and staff previously not screened for TBI or negative for TBI were screened for TBI and offered TPT. Participants with history of previous TB treatment were not screened for TBI. In addition to the above annual schedule for screening for latent TBI, active case finding using symptom criteria and molecular diagnostics was performed twice a year for all students and staff in all the schools, one round conducted simultaneously with the screening for latent TBI and the second round conducted after 6 months. All TB cases detected during the screenings for latent TBI and active disease, and during the year, were recorded. Because of the school-wide screening, contacts of recent TB cases were automatically screened for TB disease. Screenings were timed such that TPT administration and completion happened at the schools before the start of schools’ annual winter breaks. Screening and diagnosis of TBI and TB disease and TPT implementation were carried out according to a prespecified algorithm (S1 Fig) [9]. Each child or staff member was interviewed by project staff using a standardized questionnaire, evaluated by a medical officer, and screened for TBI using tuberculin skin testing (TST) with 5 TU of purified protein derivative RT23 (Span Diagnostics, Surat, India), read after 2–3 days. TST-positive (≥10 mm) individuals had a chest X-ray, following which TPT was offered to those without evidence of active TB disease. Participants were evaluated for active TB using WHO symptom criteria (cough, fever, night sweats, weight loss, tiredness, chest pain), chest X-ray, Xpert MTB/RIF IV assay (Cepheid, Sunnyvale, California), and TB culture. Active TB was treated according to Tibetan and Indian guidelines with 2 months of daily isoniazid, rifampin, ethambutol, and pyrazinamide followed by 4 months of daily isoniazid, rifampin, and ethambutol for all drug-susceptible TB. According to India’s National Strategic Plan for Tuberculosis Elimination, TPT is encouraged for high-risk groups including child pulmonary TB contacts, people living with HIV, immunosuppressed patients, and high-risk adult contacts [10].

### TPT

All participants detected with TBI were offered TPT, initially with 3 months of daily isoniazid and rifampicin (3HR) and later in the study with 4 months of daily rifampicin (4R), due to side effects encountered by children and staff with 3HR. Participants with exclusive contact with MDR-TB cases were not given TPT but underwent monitoring according to WHO guidelines. Participants with chronic hepatitis B virus (HBV) infection had liver function testing (serum bilirubin, alanine transaminase [ALT], and aspartate aminotransferase [AST]) and abdominal ultrasonographic exam to rule out liver parenchymal disease, according to the local standard of care. Liver function testing was not done for participants without HBV infection. HBV infection status was available with the school clinic as a part of their routine wellness check. Project staff conducted monthly follow-up of the TPT recipients. Hepatotoxicity was determined by the clinician based on development of (1) clinical symptoms of nausea, vomiting, loss of appetite, tiredness, upper abdominal pain, or jaundice; (2) elevated liver enzymes (≥5 times the upper limit of normal if asymptomatic or ≥3 times the upper limit of normal if symptomatic); and (3) subsidence of symptoms and laboratory values following treatment discontinuation or withholding. TPT was temporarily withheld in the event of hepatoxicity or other adverse drug reactions, as decided by the clinician, and then restarted, as appropriate. Those who had intolerance to 3HR were offered 4R, and those developing intolerance to 4R were offered 6 months of isoniazid. Therapy modification or premature termination was recommended by the physician in consultation with the participant, school nurse, home mothers, and parents.

### Statistical analysis

Data from individual schools were separately maintained year-wise in secure databases. Demographic, clinical, treatment, and laboratory data of schoolchildren and staff members from enrollment to December 31, 2019, were available. A participant ID was issued to each participant and served as the unique identifier for the longitudinal data. Baseline characteristics of schoolchildren and staff members stratified by TPT receipt were separately described. Annual incidence of TB disease was calculated as total number of new TB cases in students for the year divided by total number of students registered in the school. In addition to incidence proportion, incidence rates—overall and stratified by TPT receipt—were calculated as TB disease development over child-years at risk in the specified time. The data were analyzed according to a prospectively specified statistical analysis plan (S1 Appendix).

For the primary analysis, we assessed whether schoolchildren who received TPT had a lower risk of developing active TB as compared to schoolchildren who did not receive TPT. Participants were followed from the time of enrollment into the project and censored at the earliest of the following: (1) first development of active TB disease, (2) graduation or exit from school, or (3) December 31, 2019. Hazard ratios were calculated using the Cox proportional hazard regression model, with 95% confidence intervals using the Huber–White robust estimator of the variance. Person-time was calculated as the total follow-up time in years for a person from enrollment into the project until censoring. Persons who developed active TB ceased to contribute person-time for the calculation of incidence rates or Cox regression. Persons who developed active TB were censored on the date of diagnosis. This is an open cohort, and children graduate from high school after grade 12. Otherwise, the dropout or loss to follow-up rate is less than 5%. In secondary analyses, we compared the hazard of TB disease for schoolchildren based on TPT receipt restricting the analysis to schoolchildren with recent TB contact, and compared the 2 TPT regimens, 3HR versus 4R. Recent contact was defined as contact with a TB case in the classroom or dormitory in school, or with a family member at home, in the previous 2 years. Age, TPT receipt status, TST positivity, TB contact history, and TB-related symptoms were time dependent. All models were adjusted for age and sex. In separate analyses, the hazard of TB disease development was independently modeled as a function of each of age, sex, enrollment year, contact history, TST positivity, TST conversion, and TB-related symptoms, separately. Prompted by feedback from peer reviewers, we further assessed the risk of TB disease or infection based on (1) whether TB exposure happened at school or at home, (2) contact with drug-resistant TB, and (3) bacille Calmette–Guérin (BCG) vaccination status. The relationship between participant characteristics or risk factors and risk of TBI among schoolchildren was modeled using logistic regression, calculating Huber–White robust standard error to account for clustering in the data. Insufficient follow-up years and disease outcomes precluded investigation of the effect of TPT in adult staff members. Risk of TBI for staff members as a function of the above risk factors has been previously described [9]. Data were processed and analyzed using Stata version 13.1 software (StataCorp, College Station, Texas). This study is reported as per the Strengthening the Reporting of Observational Studies in Epidemiology (STROBE) guideline (S1 STROBE Checklist).

## Results

### Demographic characteristics

Between April 1, 2017, and December 31, 2019, 6,582 schoolchildren and 807 staff members participated in the program, contributing 13,161 and 1,800 person-years of follow-up, respectively (Table 1 and S1 Table). The median follow-up time was 2.3 years for students and 2.5 years for staff members. The majority of students (76%) were enrolled in 2017. Students were equally distributed between males and females, with a median age of 13 years. Three percent of students were previously treated for TB disease, and 28% reported a TB contact within the previous 2 years. The majority of the schoolchildren (87%) were BCG vaccinated.

**Table 1:** Baseline characteristics of children and adolescents who did and did not receive TPT in Tibetan boarding schools in Himachal Pradesh, India (2017–2019).

Characteristics	All participants (*N* = 6,582)	Participants who received TPT (*N* = 1,192)	Participants who did not receive TPT (*N* = 5,390)
**Follow-up years, median (IQR)**	2.3 (1.61–2.51)	2.3 (2.29–2.61)	2.3 (1.61–2.51)
**Total person-years of follow-up**	13,161	2,164	10,997
**Enrollment of new schoolchildren**			
2017^$^	5,020 (76.3)	799 (66.7)	3,971 (73.8)
2018^@^	655 (10.0)	87 (7.3)	579 (10.7)
2019^&^	907 (13.8)	312 (26)	834 (15.5)
**Age (years), median (IQR)**	13 (10–16)	14 (11–16)	13 (10–15)
**Sex**			
Female	3,347 (50.9)	527 (44.2)	2,820 (52.3)
Male	3,235 (49.1)	665 (55.8)	2,570 (47.7)
**Place of birth**			
India	5,362 (81.5)	928 (77.9)	4,429 (82.3)
Tibet	567 (8.6)	126 (10.6)	441 (8.2)
Nepal	612 (9.3)	130 (10.9)	481 (8.9)
Bhutan	30 (0.5)	7 (0.6)	23 (0.4)
Other	11 (0.2)	1 (0.1)	10 (0.2)
Weight (kg), median (IQR)	42 (30–52)	46 (34–55)	41 (29–52)
**History of previous TB treatment**			
Previous TB treatment	191 (2.9)	0 (0.0)	191 (2.9)
Previous MDR-TB treatment, *n/N* (%)	6/191 (3.1)	0 (0.0)	6/191 (3.1)
**TB contact history in past 2 years**			
TB contact at school/home	1,843 (28.0)	498 (41.8)	1,345 (25.0)
No contact at school/home	4,739 (72.0)	694 (58.2)	4,045 (75.0)
**Received BCG vaccine**			
Yes	6,185 (94)	1,147 (96.2)	5,038 (93.5)
No	389 (5.9)	44 (3.7)	345 (6.4)

Data are *n* (percent) unless otherwise indicated.

^$^In 2017, all schoolchildren were screened for latent TB infection.

^@^In 2018, 1,122 schoolchildren were screened for latent TB infection, of which 655 were students newly joining the school and 467 were students who were already enrolled but had recent TB exposure. The whole school was not screened for latent TBI in 2018 due to resource and time constraints.

^&^In 2019, all schoolchildren who were previously not screened for latent TB infection or had tested negative (*n* = 4,382 children and adolescents) were screened for latent TB infection. Of the 4,382 students, 907 had newly joined the school and, hence, freshly enrolled in the study.

BCG, bacille Calmette–Guérin; MDR-TB, multidrug-resistant tuberculosis; TB, tuberculosis; TPT, tuberculosis preventive treatment.

### Risk of TB disease and infection

**Fig 1:**
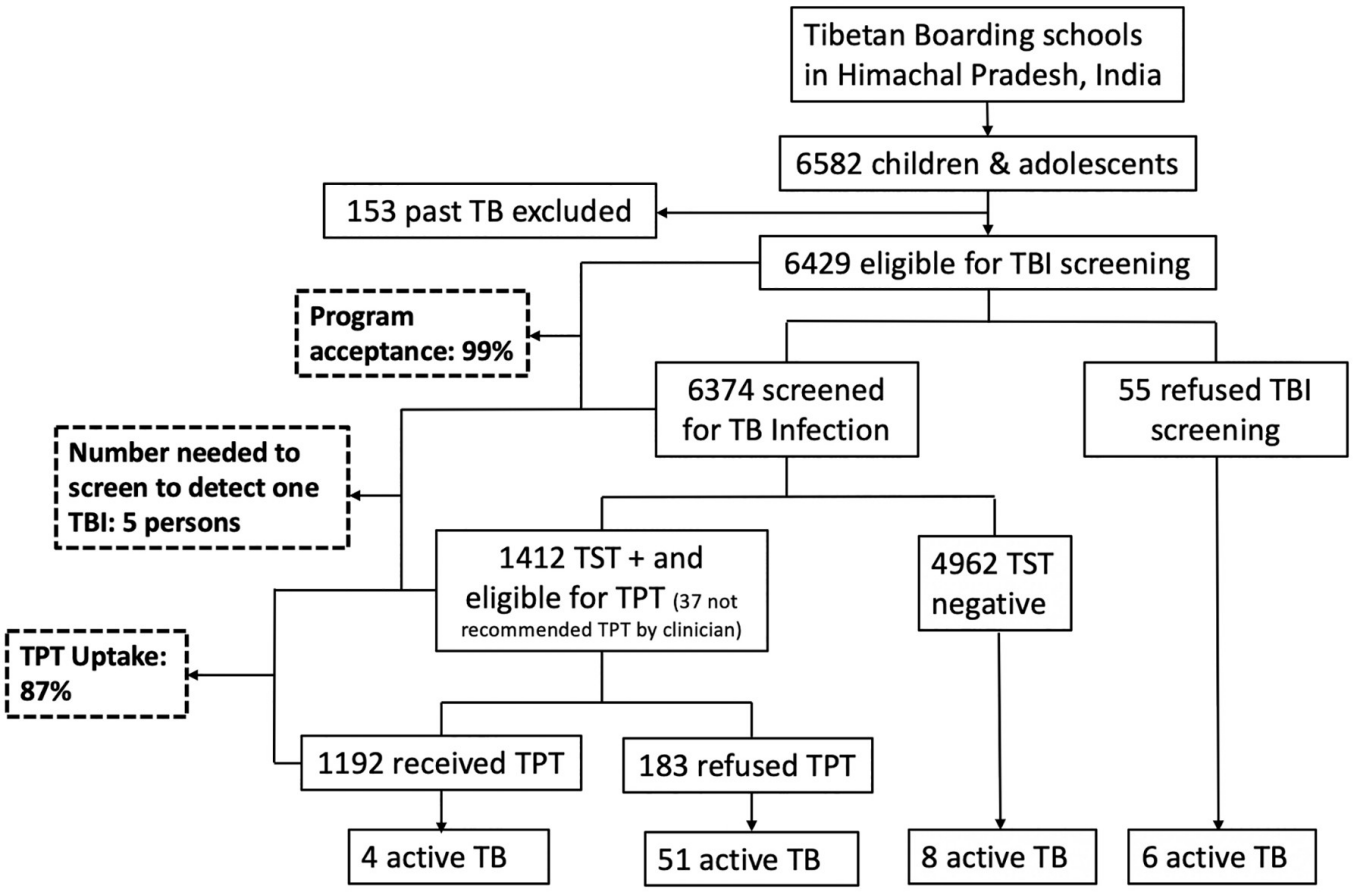
Care cascade for TBI screening and TPT in children and adolescents from 2017 through 2019. TB, tuberculosis; TBI, tuberculosis infection; TPT, tuberculosis preventive treatment; TST, tuberculin skin testing.

**Fig 2:**
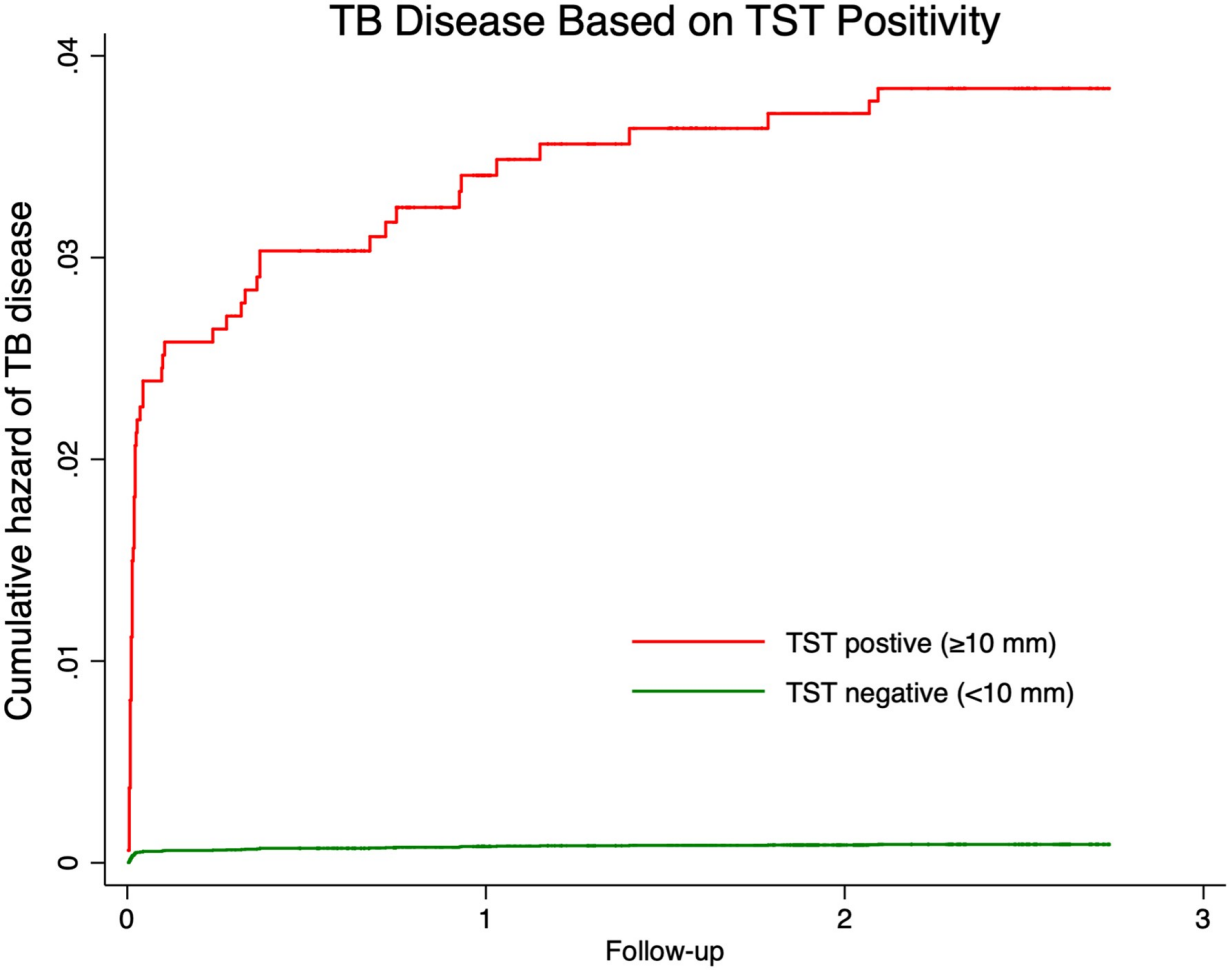
Cumulative hazard of TB disease based on TST positivity in children and adolescents from 2017 through 2019. TB, tuberculosis; TST, tuberculin skin testing.

There were 73 TB cases in total, 69 in schoolchildren and 4 in staff members (Fig 1). The median age of schoolchildren who developed TB was 16 years (Table 2). Of the 69 TB cases in students, 64 were pulmonary (60 Xpert-confirmed) and 1 was MDR-TB. Eighty percent (*n* = 55) of the TB cases in schoolchildren were TST positive, and 12% (*n* = 8) were TST negative. Fifty-nine percent (41/69) of the cases in schoolchildren did not have cough and 35% (24/69) were asymptomatic, representing a significant burden of subclinical disease (Table 3). Risk of disease increased with age (adjusted hazard ratio [aHR] 1.24; 95% CI 1.19–1.29; *p <* 0.001); recent contact history (aHR 2.55; 95% CI 1.52–4.27; *p <* 0.001); TST positivity (aHR 42; 95% CI 16.87–105.75; *p <* 0.001) (Fig 2); and TST conversion (aHR 3.00; 95% CI 1.37–6.56; *p* = 0.006) (Table 2). The risk of TB disease in children did not differ based on previous TB treatment (aHR 0.79; 95% CI 0.19–3.35; *p* = 0.748). Of 6,374 children and adolescents screened for TBI over 3 years, 1,412 (22%) were TST positive. Increasing age and male sex were associated with increased risk of TBI. Among children and adolescents who had recent exposure to TB (i.e., in the last 2 years) (*n* = 1,843), 1,610 (87%) had exposure at school, 141 (8%) had exposure at home, and 92 (5%) had exposure both at school and at home. We did not find a significant difference in the risk of TB disease or TBI based on exposure at home versus school. The risk of TBI was significantly higher for children who had exposure at both school and home (aHR 1.74; 95% CI 1.09–2.78; *p* = 0.021). BCG vaccination was not associated with any protective effect or increased risk of TBI and TB disease in the schoolchildren. There were 4 TB cases among staff members, of which 1 was extensively drug-resistant TB.

**Table 2:** Risk factors for TB disease and infection among children and adolescents (*N* = 6,582) in Tibetan boarding schools in Himachal Pradesh, India (2017–2019).

Characteristic	Detected with TB disease, *N* = 69	TB risk aHR^#^ (95% CI); *p-*value	Detected with TB infection, *N* = 1,412	TB infection risk aOR^#^ (95% CI); *p*-value
**Age (years), median (IQR)**	16 (14–17)	1.24 (1.19–1.29); *p <* 0.001	14 (11–16)	1.15 (1.13–1.17); *p <* 0.001
**Sex, *n* (%)**				
Male (*n* = 3,235)	31 (45)	0.82 (0.51–1.33); *p* = 0.422	772 (54.7)	1.33 (1.17–1.51); *p <* 0.001
Female (*n* = 3,347)	38 (55)	Reference	640 (45.3)	Reference
**TB exposure, *n* (%) or *n/N* (%)**				
Recent TB contact (*n* = 1,843)*	43 (62.3)	2.55 (1.52–4.27); *p <* 0.001	501/1,094 (46)	2.03 (1.77–2.35); *p <* 0.001
No recent TB contact (*n* = 4,739)	26 (37.7)	Reference	593/1,094 (54)	Reference
**TB exposure at school or home, *n* (%)**				
Recent exposure at school only (*n* = 1,610)^@^	35 (51)	Reference	522 (37)	Reference
Recent exposure at home only (*n* = 141)^@^	3 (4.3)	1.80 (0.52–6.18); 0.352	39 (2.8)	0.95 (0.68–1.34); *p* = 0.778
Recent exposure at school and home (*n* = 92)^@^	5 (7.2)	2.04 (0.62–6.69); 0.237	41 (2.9)	1.74 (1.09–2.78); *p* = 0.021
**Past TB treatment, *n/N* (%)**				
Previous TB (at baseline) (*n* = 153)	4/64 (6.3)	0.79 (0.19–3.35); *p* = 0.748	—	—
No previous TB (*n* = 6,420)	60/64 (93.7)	Reference	—	—
**TST status, *n* (prevalence)**				
TST negative (*n* = 4,962)	8 (160/100,000)	Reference	—	—
TST positive (*n* = 1,412)	55 (3,900/100,000)	42 (16.87–105.75); *p <* 0.001	—	—
TST not done (*n* = 208)	6 (2,880/100,000)	—	—	—
**TST conversion, *n/N* (%)**				
Always TST negative (*n* = 4,962)	8/62 (12.9)	—	—	—
TST positive (at baseline) (*n* = 1,061)	48/62 (77.4)	Reference	—	—
TST converters (*n* = 347)	6/62 (9.7)	3.00 (1.37–6.56); *p* = 0.006	—	—
**Contact with MDR-TB, *n/N* (%)**				
Yes (*n* = 106)	0/44 (0)	—	28/604 (4.5)	0.59 (0.35–0.98); *p* = 0.040
No (*n* = 1,833)	44/44 (100)	—	596/604 (95.5)	Reference
**BCG vaccine received, *n* (%)**				
Yes (*n* = 6,185)	64 (92.8)	1.01 (0.48–2.13); *p* = 0.974	1,350 (95.6)	1.01 (0.88–1.17); *p* = 0.850
No (*n* = 389)	2 (2.9)	Reference	59 (4.2)	Reference

^#^Adjusted for age and sex.

*Contact with at least 1 TB case in past 2 years.

^@^92 students had exposure at both home and school.

aHR, adjusted hazard ratio; aOR, adjusted odds ratio; BCG, bacille Calmette–Guérin; MDR-TB, multidrug-resistant tuberculosis; TB, tuberculosis; TST, tuberculin skin testing.

**Table 3:** Characteristics of children and adolescents diagnosed with tuberculosis (TB) disease in the boarding schools from 2017 to 2019.

Outcome	Children and adolescents (*N* = 69)	Staff members (*N* = 4)
**Smear positive**	13/67 (19%)	1 (25%)
**Smear negative**	54/67 (81%)	3 (75%)
**Xpert result**		
Positive	60/61 (98%)	3 (75%)
Negative	1/61 (2%)	1 (25%)
**Xpert *Mycobacterium tuberculosis* load**		
Very low	17/59 (29%)	0/3 (0%)
Low	25/59 (42%)	3/3 (100%)
Medium	16/59 (27%)	0/3 (0%)
High	1/59 (2%)	0/3 (0%)
**Rifampin resistance (Xpert)**		
Sensitive	60/61 (98%)	2/3 (67%)
Resistant	1/61 (2%)	1/3 (33%)
**Culture result**^#^		
Positive	27/63 (43%)	2 (50%)
Negative	36/63 (57%)	2 (50%)
**Drug resistance**		
Multidrug-resistant TB	1 (1%)	0 (0%)
Extensively drug-resistant TB	0 (0%)	1 (25%)
**Disease site**		
Pulmonary only	64 (93%)	4 (100%)
Extrapulmonary only	4 (6%)	0 (0%)
Pulmonary and extrapulmonary	1 (1%)	0 (0%)
**TB-related symptoms**		
No cough	40 (58%)	2 (50%)
Cough	29 (42%)	2 (50%)
Cough for <2 weeks	17 (25%)	1 (25%)
Cough for ≥2 weeks	11 (16%)	1 (25%)
Productive cough	24 (35%)	2 (50%)
No TB symptoms	23 (33%)	2 (50%)
Any TB symptom	46 (67%)	2 (50%)

Data given as *n/N* (%) or *n* (%).

^#^Culture was not performed for Xpert-negative samples except in 4 individuals with clinical suspicion of TB. Culture was performed for Xpert-positive cases.

### TPT completion and safety

Of 6,374 children and adolescents screened for TBI over 3 years, 1,412 were TST positive, of whom 1,192 received TPT; the physician recommended that 37 should not receive TPT and 183 refused TPT (Fig 1). There were 41 children with chronic HBV infection who were found to have latent TBI. Of these 41 children with TB and HBV coinfection, 11 (27%) did not receive TPT; they either refused treatment or the clinician did not recommend TPT. None of the children with HBV infection who underwent liver function testing before initiation of TPT had elevated transaminases. Few of the children were receiving antiviral treatment for chronic HBV at the time of screening. While children and adolescents who did (*n* = 1,192) and did not (*n* = 5,390) receive TPT were mostly similar in characteristics, TB exposure was higher in TPT recipients (42% versus 25%). Of the 1,192 students and 125 staff members who received TPT, TPT completion information was available for 1,043 students and 115 staff; the remaining were undergoing TPT at the time of analysis (S2 Table). Of 1,043 students and 115 staff who received TPT, 98% (1,025) and 100% completed TPT, respectively. TPT completion was similar between 3HR and 4R for schoolchildren and staff. For both children (5–14 years) and adolescents (10–19 years), the prevalence of any side effects related to various body systems was significantly lower for 4R (range 0%–4%) than for 3HR (range 0%–14%) (S3 Table). Prevalence of hepatotoxicity was less than 0.5% (*n* = 3) for both 3HR and 4R regimens in schoolchildren.

### TB incidence following TPT

**Fig 3:**
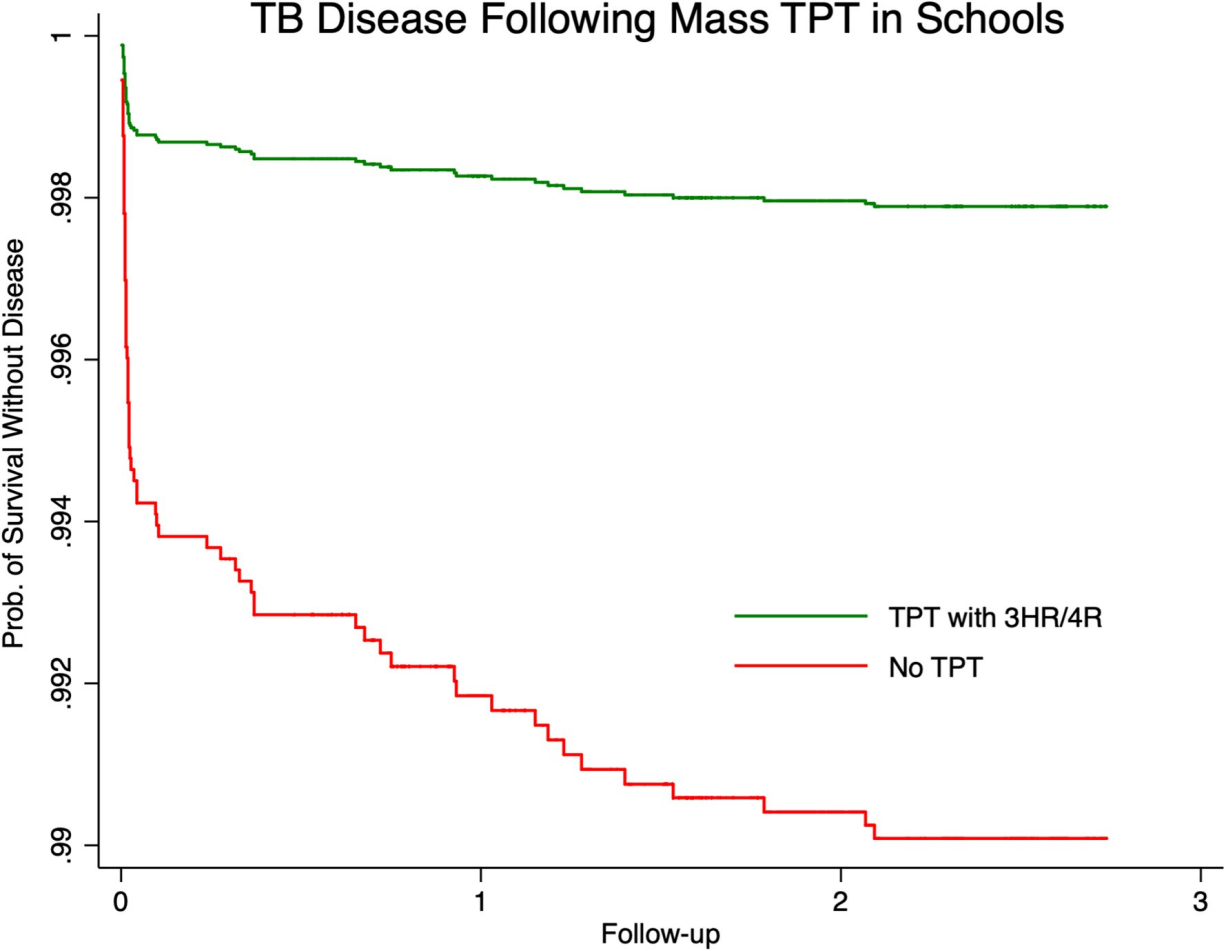
Risk of TB disease for children and adolescents who did and did not receive TPT. 3HR, 3 months of daily isoniazid and rifampin; 4R, 4 months of daily rifampin; TB, tuberculosis; TPT, tuberculosis preventive treatment.

The average annual incidence rate for schoolchildren over 13,161 person-years of follow-up was 524/100,000 (95% CI 414–663/100,000) person-years (Table 4). Among persons receiving TPT (2,164 person-years), 4 participants developed TB disease, and among persons not receiving TPT (10,997) person-years), 65 participants developed TB disease, yielding average annual TB incidence rates of 185/100,000 (95% CI 69–492/100,000) person-years and 591/100,000 (95% CI 464–754/100,000) person-years, respectively (*p* = 0.009). In multivariate analysis adjusted for age and sex, participants receiving TPT had a 79% lower hazard of TB disease development compared to those who did not receive TPT (aHR 0.21; 95% CI 0.07–0.69; *p* = 0.010) (Fig 3). The preventive effect was more pronounced for participants with a recent contact history, with incidence rates of 125/100,000 (95% CI 18–890/100,000) person-years for those receiving TPT versus 1,643/100,000 (95% CI 1,190–2,267/100,000) person-years for those who were not treated (aHR 0.07; 95% CI 0.01–0.42; *p* = 0.004). Of all the children and adolescents who received TPT, 4 developed TB disease, and they had all received 3HR. No 4R recipients developed TB disease. The TB that occurred in the 4 TPT recipients was drug susceptible. The children who developed TB post-TPT were adherent to treatment. The mean number of days to TB development after TPT completion was 302 days (SD 118 days). The TB incidence rate difference for use of 3HR versus 4R (0.00222/100,000 person-years; 95% CI 0.00004–0.00439/100,000) was not statistically significant (*p* = 0.384); however, the follow-up time was short for 4R (343 person-years). Four staff members developed TB disease over 1,800 person-years of follow-up (annual incidence rate 256/100,000 person-years; 95% CI 96–683/100,000); 1 case was extensively drug-resistant TB. No staff who developed TB disease had received TPT. We calculated a number needed to screen of 5 persons to detect 1 TBI, number needed to treat (NNT) with TPT of 248 persons for all schoolchildren to prevent 1 TB disease, and NNT of 64 for schoolchildren with recent TB contact.

**Table 4:** TPT and risk of TB disease development in children and adolescents in Tibetan boarding schools in India (2017–2019).

Exposure group	Person-years of follow-up	Total cases	Incidence rate (95% CI) per 100,000 person-years	Adjusted hazard ratio* (95% CI); *p-*value	IRR^@^ (95% CI); *p-*value	NNT to prevent 1 TB disease^#^
**Children and adolescents**	13,161	69	524 (414–663)	—	—	—
**Overall risk**						
No TPT	10,997	65	591 (464–754)	Reference	Reference	
Received TPT	2,164	4	185 (69–492)	0.21 (0.07–0.69); *p* = 0.010	0.23 (0.07–0.80); *p* = 0.021	254
**Recent contacts only**^$^						
No TPT	2,252	37	1,643 (1,190–2,267)	Reference	Reference	
TPT (3HR/4R)	797	1	125 (18–890)	0.07 (0.01–0.42); *p* = 0.004	0.06 (0.01–0.42); *p* = 0.005	64
**TPT regimen**						
Received 3HR	1,805	4	222 (83–591)	0.00222^&^ (0.00004–0.00439); *p* = 0.384	—	—
Received 4R	342	0	0			

*Cox proportional hazard model adjusted for age and sex.

^@^IRR adjusted for age and sex.

^#^NNT to prevent 1 disease calculated as 1/(Incidence_untreated_ *−* Incidence_treated_).

^$^Analysis restricted to participants reporting history of TB contact in previous 2 years.

^&^Incidence rate difference with 95% CI calculated.

3HR, 3 months of daily isoniazid and rifampin; 4R, 4 months of daily rifampin; IRR, incidence rate ratio; NNT, number needed to treat; TB, tuberculosis; TPT, tuberculosis preventive treatment.

### Impact on TB burden

**Fig 4:**
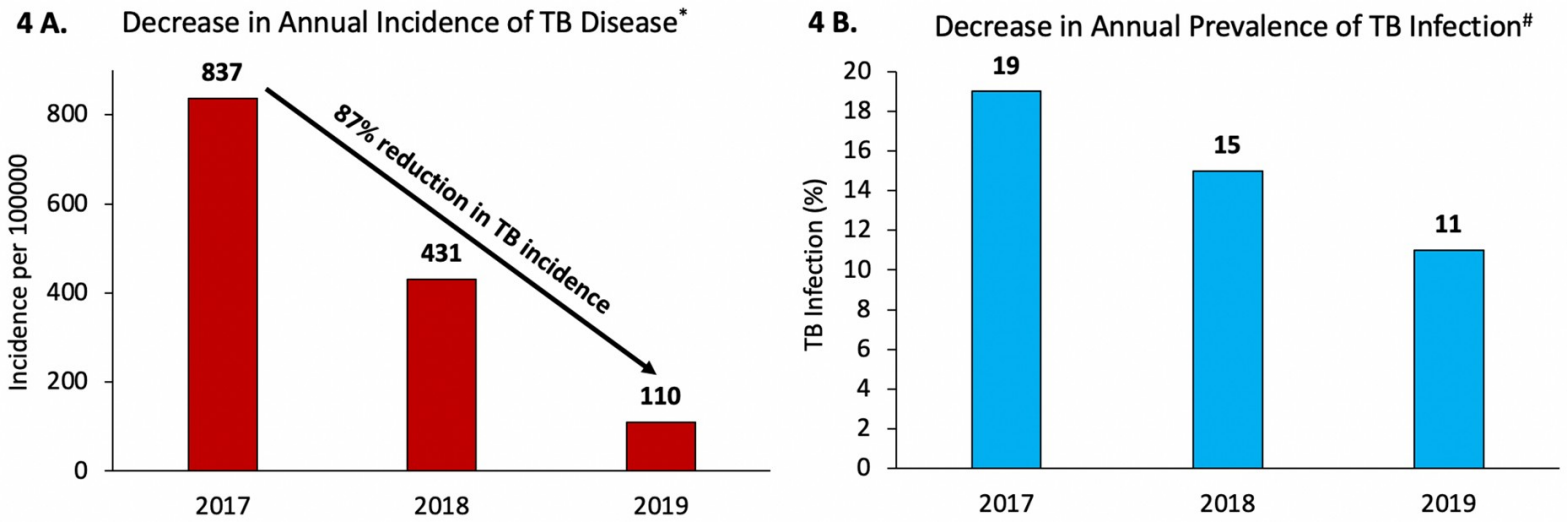
Decline in tuberculosis (TB) burden in children and adolescents in the Tibetan boarding schools in India from 2017 through 2019. (A) Decrease in annual incidence of TB disease. (B) Decrease in annual prevalence of TB infection. *Incidence of annual TB disease represents the proportion of new episodes of TB cases per 100,000 students per year among all students enrolled in the schools (Table 5). ^#^Prevalence of annual TB infection represents the proportion of students testing positive for tuberculin skin test per year among all students at the schools who were not tested previously or had tested negative previously.

Among schoolchildren who were annually screened for latent TBI, the prevalence of latent TBI in the Tibetan boarding schools decreased from 19% (913/4,860) in 2017 to 15% (124/827) in 2018 to 11% (383/3,608) in 2019 (Fig 4; Table 5). The overall incidence of TB disease in schoolchildren declined from 837/100,000 person-years (42/5,020) in 2017 to 431/100,000 person-years (23/5,339) in 2018 and 110/100,000 person-years (5/4,566) in 2019, an 87% decrease in incidence from 2017 to 2019.

**Table 5:** Decrease in annual incidence and prevalence of tuberculosis (TB) disease and infection, respectively, in children and adolescents in Tibetan boarding schools in India (2017–2019).

Year	Total student population	Total number of TB cases	Incidence of TB disease per 100,000 (95% CI)	Decrease in TB disease incidence* (%)	Total number of TB infections (*n/N*)	Prevalence of TB infection, percent (95% CI)	Decrease in TB infection prevalence* (%)
2017^#^	5,020	42	837 (604–1,129)	Reference	913/4,860	19 (18–20)	Reference
2018^#^	5,339	23	431 (273–646)	51	124/827	15 (13–18)	21
2019	4,566	5	110 (36–255)	87	383/3,608	11 (10–12)	42

*Percent decrease calculated as (Incidence_*i*th year_ − Incidence_*i*th year*+n*_)/Incidence_*i*th year_, where *i* = 2017 and *n* = 1, 2,….*N*.

^#^One child had TB in 2017 with a recurrent episode in 2018. Both the episodes were included as incident cases in 2017 and 2018.

## Discussion

Motivated by the United Nations Sustainable Development Goals and India’s commitment toward ending TB, we undertook an initiative to eliminate TB in Tibetan refugee children by implementing a comprehensive screening and treatment program for both TBI and TB disease in a network of boarding schools. Between 2017 and 2019, we observed an average annual incidence rate of TB of 524/100,000 person-years in schoolchildren and 256/100,000 person-years in adult staff members in the Tibetan boarding schools in Himachal Pradesh. In the initial year of screening in 2017, an incidence of TB disease of 837/100,000 person-years and prevalence of TBI of 19% were observed in schoolchildren. After 3 years of program implementation, TB incidence declined by 87%, and prevalence of TBI declined by 42%, in children and adolescents in the schools. None of the staff members who received TPT developed TB disease while 4 staff members who did not receive TPT developed TB disease. The reductions in TB associated with 3HR and 4R TPT regimens were similar. TPT implementation had the greatest value in preventing TB cases in the first 2 years. Our work demonstrates that population-wide active case finding and TPT implementation using a community-based approach can effectively control TB in high-transmission settings where the chances of reinfection are high. Since 2011, active case finding of TB has been annually carried out in the Tibetan boarding schools under the Department of Health of the CTA. However, despite the effort, TB case rates have remained high over the years. Mass screening and TPT—by limiting disease progression, reactivation, and transmission—have driven this exponential reduction in TB rates. The successful control in this population is comparable to the reduction in TB incidence (>75%) achieved in Peru in 1985–1986 and during the community-wide isoniazid prophylaxis trial in the Bethel area in Alaska in the 1960s [11,12].

To date, very few studies have assessed TPT efficacy or effectiveness in children ≥ 5 years or in adolescents [13,14]. The reduction in TB disease risk of 79% associated with TPT use in our child and adolescent population is similar to or better than that observed in independent IPT trials in children 2 months–15 years (67%) in the Bethel area in Alaska in 1962 [15]; children 5–15 years (60%) in France between 1959 to 1969 [16]; children 0–15 years (60%) in the US, Canada, and Mexico in 1961 [17]; and children 0–15 years (62%) in Kenya in 1965 [18]. Applying the TB incidence of 837/100,000 person-years observed in the initial year in 2017 to the average annual student population of 5,000, 126 TB cases (42 annually) could have resulted over 3 years in the absence of the ZTBK program instead of the observed 69 cases. As such, 56 schoolchildren were spared from developing TB disease over 3 years. Despite less follow-up time for staff members, TPT showed protection in adult staff members as well; none of the adult staff who received TPT (*n* = 240 person-years) developed active TB whereas 4 TPT non-recipients (*n* = 1,560 person-years) developed active TB. The long-term benefit of TPT has been debated in the past. Investigators in South Africa observed waning of protection in a mass IPT intervention study in adult gold miners in South Africa [19,20]. This may be partly attributable to older age, higher HIV prevalence, high rates of silicosis, and constant interactions with a wider community harboring undiagnosed TB cases. In this study, we observed a sustained benefit over 3 years. The closed nature of the boarding schools, with their physical boundaries, restricted visitors, and less frequent interactions with the community may have helped to sustain the benefit of the TPT for our population. The overall reduction in TB rates observed in our study, including in those who did not receive TPT, suggests a herd benefit to other students, staff, and members of community.

Prevalence of recent TB exposure was high for schoolchildren (28%) and staff (13%). Three percent of schoolchildren and 19% of staff were treated for active TB in the past, reflecting a high disease burden and transmission in the community. In children and adolescents, TB risk was 42-fold higher for those with positive TST during the latest screening (*p <* 0.001). Of child and adolescent participants who had at least 1 longitudinal follow-up, 10% (347/3,643) converted from TST negative to positive over the 3-year period, reflecting an approximately 3.3% annual rate of acquiring new infection. The annual rate of acquiring new infections was 8% in the Bethel area in 1957, in keeping with its higher TB burden (annual incidence of approximately 1%) [21]. We observed a 3-fold higher disease risk in TST converters and 93% risk reduction from TPT in recent TB contacts. These findings underscore the importance of targeting recent TB contacts for preventive treatment in general. This is also evident from the lower number of schoolchildren who would need to be treated to prevent 1 TB disease in recent TB contacts (NNT = 68) as compared to that for the general population of schoolchildren (NNT = 253). Due to an insufficient number of outcomes of TB disease, we could not assess the rationale for TPT implementation in children ≥5 years with TB exposure without doing TST. With accumulated person-time, we intend to assess this in the future. Currently, WHO recommends TPT for child TB contacts <5 years of age regardless of TST status. Data from prevalence surveys across several communities in Africa have shown a high prevalence of TB disease (18.5%) among adults who were previously treated for TB [22]. We observed a similar prevalence of previous TB of 25% (1/4) in adults, although the sample size is small for adults in our study. For children and adolescents in our study, the prevalence of previous TB was 6.3%. We did not find a significant relationship between previous TB history and the risk of TB disease in children and adolescents.

Globally, the number of people with recent TBI is estimated at 56 million, as compared to the nearly 2 billion total people with latent TBI, making recent contacts a practically targetable population for TPT [23]. For intervention by active case finding alone, the number of people needed to screen to prevent 1 TB disease was calculated to be 1,002 in 1 study [24]. The data on TB rates in recent contacts and TST converters suggest ongoing TB transmission in schoolchildren, supporting the need to study TB transmission dynamics for appropriate public health intervention.

As a result of side effects to the 3HR TPT regimen reported by participants during the initial phase, the regimen was switched to 4R starting in March 2018. Tiredness, sleepiness, dizziness or headache, nausea, and epigastric discomfort were the more common side effects faced by the participants who received 3HR (S3 Table). The side effects were more prevalent in adolescents than in children less than 10 years old. We observed significantly fewer side effects associated with 4R as compared to 3HR for both children (5–14 years) and adolescents (10–19 years). No participant had a serious adverse event that required hospitalization. Risk of hepatotoxicity was very low (<0.5%) for both the TPT regimens. We observed high treatment completion (>95%) for both 3HR and 4R as a result of effective community engagement and mobilization, the dedication and hard work of program staff and school nurses, supervision by the home mothers, and the responsibility shouldered by individual participants. Although the study was not randomized for the comparison, we compared the 3HR and 4R regimens, and the difference in incidence rate reduction associated with the 3HR regimen compared to the 4R regimen was not statistically significant (95% CI 0.00004–0.00439/100,000 person-years; *p* = 0.384). Given the high completion rate, and better safety profile and similar reduction of TB as 3HR, 4R likely presents an effective treatment regimen for wide TPT implementation, and especially in adolescents and young adults, in whom isoniazid-related toxicity rates are higher.

A limitation of our study is that the reduction in TB incidence could be influenced by a secular trend in TB incidence during the study period. To test this possibility, we adjusted the hazard model (dependent variable: TB disease; independent variable: TPT receipt) for year of enrollment in addition to age and sex. The risk reduction from TPT use remained unchanged (aHR 0.21; 95% CI 0.07–0.68; *p* = 0.009) after this additional adjustment for calendar year. We considered the possibility that the reduction in TB disease in the second and third years could be partly due to a survival bias whereby schoolchildren having greater risk factors had developed TB disease earlier in the project period, resulting in an apparent reduction in incidence in later years. However, had there been no overall reduction in TB burden in the schools, these same children would also be at risk of reinfection and recurrence in the later years due to ongoing transmission. Therefore, it stands to reason that the reduction in TB incidence was largely the impact of our comprehensive program. Additionally, the global lack of a diagnostic tool prevented us from assessing the risk of TB reinfection for individuals who completed TPT after a positive test for TBI. We are unable to assess the reduction of TB disease as a result of TPT implementation in adult staff members due to a limited sample size. Inadequate exposure time for individual TPT regimens also precluded us from confidently comparing the effectiveness and safety of the regimens. In implementing this population-level initiative, some of the operational challenges we faced related to coordinating X-rays for children outside of schools and ensuring their safety, the need to perform gastric aspirations, especially given the high rate of subclinical disease, the inability to screen schools in parallel due to limited resources and program staff, coordinating activities vis-à-vis school schedule, and the lack of operational guidelines for implementation of TPT. These and additional challenges have been described in more detail previously [9].

Despite clinical studies and mathematical models showing the efficacy, effectiveness, and impact of TPT [25–27], its implementation has been neglectfully suboptimal [1]. Our study sheds light on various aspects of TPT implementation including screening strategies, treatment effectiveness, choice of regimens, and safety profiles that could inform national and global guidelines, specifically for children and adolescents. For the first time, to our knowledge, the TB epidemic curve is bent for this vulnerable refugee children population, setting the community on a path to TB elimination, and presenting a model for communities nationally and globally.

## Supporting information

S1 Appendix Project design and analysis plan. (DOCX)

S1 STROBE Checklist Checklist for Strengthening the Reporting of Observational Studies in Epidemiology (STROBE). (DOCX)

S1 Fig Algorithm for tuberculosis screening and preventive treatment for children and adults in the Tibetan boarding schools in India. (TIF)

S1 Table Baseline characteristics of adult staff members who did and did not receive tuberculosis preventive treatment (TPT) in Tibetan boarding schools, Himachal Pradesh, India (2017–2019). (DOCX)

S2 Table Treatment completion among schoolchildren and staff who were receiving tuberculosis preventive treatment using either 3 months of isoniazid and rifampin or 4 months of rifampin. (DOCX)

S3 Table Side effects associated with tuberculosis preventive treatment (TPT) regimens consisting of 3HR (3 months of daily isoniazid and rifampin) or 4R (4 months of daily rifampin) in children and adolescents. (DOCX)

## Abbreviations

3HR: 3 months of daily isoniazid and rifampicin
4R: 4 months of daily rifampicin
aHR: adjusted hazard ratio
BCG: bacille Calmette–Guérin
CTA: Central Tibetan Administration
HBV: hepatitis B virus
MDR-TB: multidrug-resistant tuberculosis
NNT: number needed to treat
TB: tuberculosis
TBI: tuberculosis infection
TPT: tuberculosis preventive treatment
TST: tuberculin skin testing
ZTBK: Zero TB Kids
