# Supplementary material for: Risk of developing active tuberculosis following tuberculosis screening and preventive therapy for Tibetan refugee children and adolescents in India: An impact assessment
Source: PLoS Med. 2021 Jan 19;18(1):e1003502. doi: 10.1371/journal.pmed.1003502 (PMC7853467; doi:10.1371/journal.pmed.1003502)
Supplement: S1 Appendix — (DOCX) [file pmed.1003502.s001.docx]

**Zero TB in Tibetan Kids Living in India**

**Moving from TB Control to TB Elimination**

**A Collaborative Initiative of the Johns Hopkins University and Tibetan Delek Hospital**

**Project Design and Analysis Plan**

*(Excerpted from project protocol version 0.1 dated Feb 9, 2017 and project proposals)*

**Key Persons**

Richard E Chaisson

Kunchok Dorjee

Tsetan D Sadutshang

Sonam Topgyal

**Table of Contents**

**List of Abbreviations** 3

**Summary** 4

**1. Background Information and Scientific Rationale** 6

1.1 Background Information 6

**2. Project Objectives** 6

2.1 Primary 6

2.2 Secondary 6

**3. Project Design 6**

3.1 Project Activities 7

3.2 Project Phases 10

3.3 Project Population 10

3.4 Endpoints 11

3.5 Data collection and management and confidentiality………………………………………………………………..11

3.6. Statistical Analysis Plan……………………………………………………………………………………………………………..11

3.7 Timeline 12

**4. Project Risks and Benefits** 12

4.1 Potential Risks 12

4.2 Known Potential Benefits 13

4.3 Risk/Benefit Ratio 13

**5. Selection and Enrollment** 13

5.1 Eligibility Criteria 13

5.2 Exclusion Criteria 13

**8. References** 13

**List of Abbreviations**

| ACF | Active Case Finding |
| --- | --- |
| CTA | Central Tibetan Administration |
| DIIR | Department of Information and International Relations |
| DOH | Department of Health |
| IEC | Independent or Institutional Ethics Committee |
| IRB | Institutional Review Board |
| INH | Isoniazid |
| IPT | Isoniazid Preventive Therapy |
| JHU-CTR | Johns Hopkins University Center for TB Research |
| MDR | Multi-drug resistant [TB] |
| PI | Principal Investigator |
| QA | Quality Assurance |
| QC | Quality Control |
| QM | Quality Management |
| RNTCP | India’s Revised National Tuberculosis Control Programme |
| SID | Study identification number |
| TB | Tuberculosis |
| TCV | Tibetan Children’s Village |
| TST | Tuberculin Skin Test |
| XDR | Extensively drug-resistant [TB] |

**Summary**

| **Title:** | Zero TB in Tibetan Children Living in India |
| --- | --- |
| **Abbreviated Title:** | Zero TB Kids |
| **Objectives:** | Primary   - Timely identification and treatment of prevalent TB cases - Identification and preventive therapy for latent TB infection with recent exposure history - Measurable reduction of annual TB case rate eventually leading onto to elimination   Secondary   - Implement a system for uniform TB reporting and surveillance under the CTA-DOH - Improved system of communication between school health centers and the DOH hospitals/clinics - Heightened TB awareness in the population and involvement of community leaders and members in relevant project activities - Education and training to physicians, nurses and community health workers |
| **Study Design:**  **Study Population and Sample Size:** | Quality improvement/implementation project for active TB case finding and TB preventive therapy in schoolchildren  12,000 children |
| **Number of Sites:** | 16 schools |
| **Study Duration:** | Approximately 5 years |
| **Subject Participation Duration:** | Annual screening |
| **Project Design:** | We propose to develop a model TB care quality improvement program in conjunction with the Central Tibetan Administration Department of Health that will include sustainable, transferrable systems for health education and environmental interventions, active case-finding in schools, proper treatment of drug susceptible and resistant TB disease, and preventive therapy for children with latent TB infection. Advocacy and community mobilization including involvement by spiritual leaders, as well as education and training will be core components of the program. After an implementation period of two years in Himachal Pradesh and Uttrakhand, the project will be rolled out to include Tibetan children living schools and monasteries in South India. This project is being developed with the goal to eliminate TB in the Tibetan children population in India. |
| **Endpoints:** | Primary Outcome Measures  - TB rates measured over time during active case finding campaigns - TB preventive therapy initiation and completion rates |

# Background Information and Scientific Rationale

## BACKGROUND INFORMATION

The prevalence of TB is extremely high in the Tibetan population living in exile in India [1, 2]. TB is the single most important public health problem in the Tibetan community. According to data from the Tibetan TB Control Programme, the incidence rate of TB among Tibetans living in India in 2014 was 467 per 100,000 people, whereas the incidence rates in 2014 in India is 167/100,000 people. While poverty, unemployment, socio-cultural and language incompatibility, and inadequate access to care have significantly contributed towards a high burden of TB among Tibetans in the past several decades, currently, living in congregate settings such as boarding schools and monasteries, household transmission, and delayed diagnosis in the schools are likely the more important contributory factors. In 2011, JHU CTR secured a WHO TB REACH grant to conduct active case finding of TB among Tibetan students and monks/nuns living in the schools and monasteries across India. JHU CTR collaborated with the Delek Hospital to carry out the TB REACH project. Under this project, between September 2011 and March 2013, 27,714 persons were screened for symptoms of TB in 21 Tibetan schools and 36 Tibetan monasteries across India. This includes 436 newly arrived refugees from Tibet. There were 96 cases of TB detected during the ACF project; 47 cases were among students, yielding a prevalence rate of 394/100,000 students. The overall prevalence of TB in the entire population screened in the TB REACH project was 346 per 100,000 people. The prevalence rate was higher in the monastic population in Mundgod (486/100,000 persons) and in Uttrakhand (721/100,000 persons).

1. **STUDY OBJECTIVES**
   1. **PRIMARY OBJECTIVES**

- Timely identification and treatment of prevalent TB cases
- Identification of and preventive therapy for latent TB infection with recent exposure history
- Measurable reduction of annual TB case rate eventually leading onto to elimination
  1. **SECONDARY OBJECTIVES**
- To implement a system for uniform TB reporting and surveillance under the CTA-DOH
- Improved system of communication between school health centers and the DOH hospitals/clinics
- Heightened TB awareness in the population and involvement of community leaders and members in relevant project activities
- Education and training to physicians, nurses and community health workers

1. **PROJECT DESIGN**

The overall goal of this project is to reduce TB and MDR-TB case rates in children enrolled in Tibetan schools and monasteries through implementation of a model system of TB care. The long-term goal of implementation of this model system of care would be to eliminate TB among Tibetan children living in India. The proposed model TB control program will constitute sustainable systems for diagnosis and early case detection, treatment of drug susceptible and resistant disease, provision of preventive therapy for LTBI, surveillance/reporting, and education, training and community mobilization. The project staff will be trained before implementation on 1) Overall layout of the 'Zero TB in Tibetan Kids' project, 2) Active Case Finding and Contact Investigation, 3) Latent TB Infection and TB Preventive Therapy, 4) Monitoring and management of adverse drug events from TB preventive therapy, and 5) Tuberculin Skin Test – Background and Administration. All activities will build upon the existing standard of care, with specific attention in the following areas:

- **Find TB**: Active TB case finding, contact investigation, and TB diagnosis through innovative strategies.
- **Treat TB:** Proactive approach with use of scientifically approved newer anti-TB medicines as necessary while following national and international guidelines.
- **Prevent TB:** Community education, infection control, LTBI screening, and use of suitable TB preventive therapy.
- **Track TB:** Uniform TB reporting and surveillance

**Figure 1**. A conceptual framework for TB control and elimination children


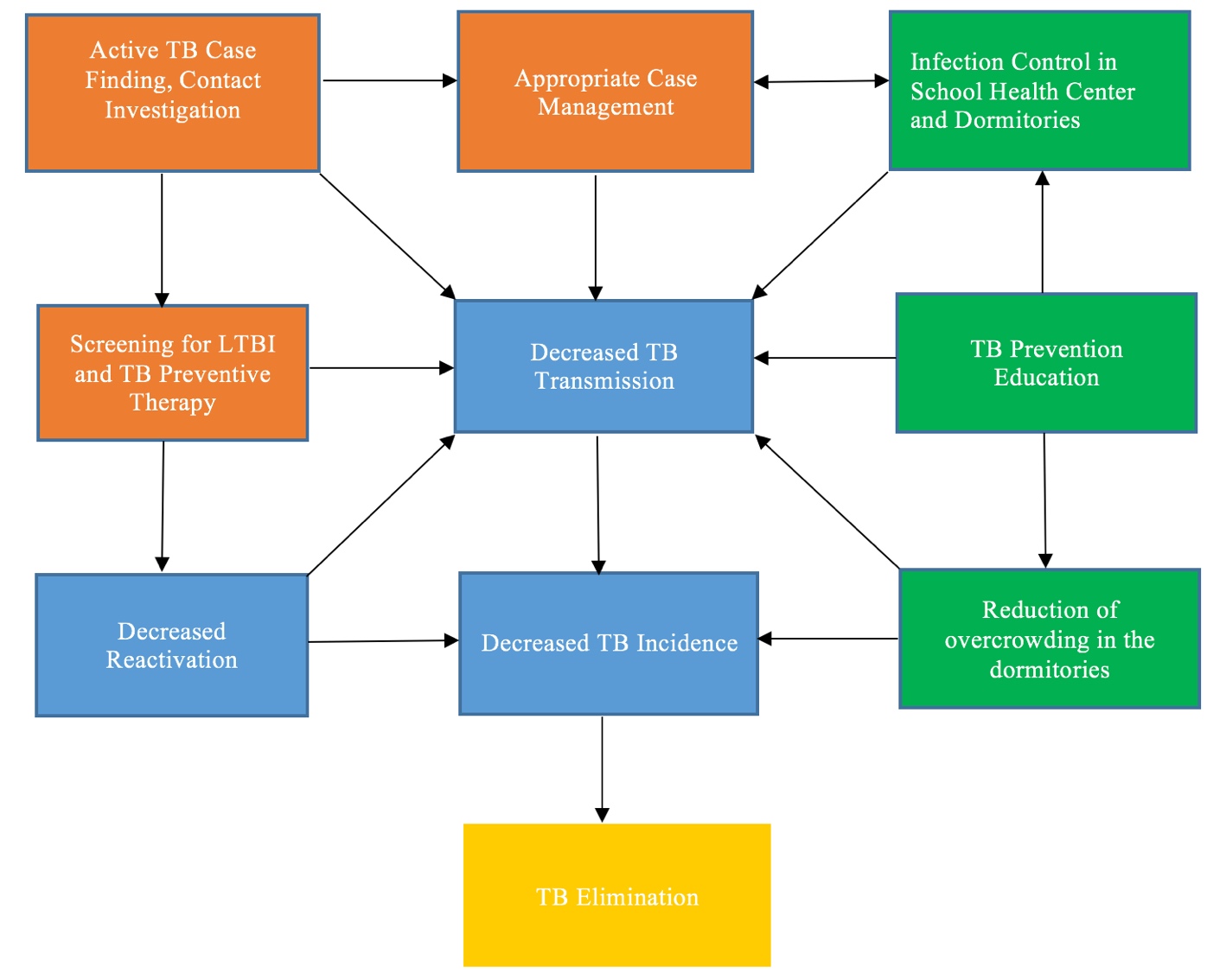


### Project Activities

### Community Awareness

The project shall be commenced with an initial mass community awareness campaign to be launched via a social media platform such as Facebook, a dedicated program through a Voice of America Television’s Tibetan service, and through a Tibet Online Television run by the CTA Department of Information and International Relations (DIIR). An education and training core will provide services to community health workers, physicians and nurses, and the community. “Toolkits” will be developed, piloted, and implemented in order to maximize education and training capacity during the intervention and to sustain education and training after the intervention. Participation of the class teachers and the home-mothers caring for the students in the dormitories shall be sought. The support of spiritual figures in generating awareness in the community regarding the activities and goals of the project would be important and shall be sought. Community shall be educated regarding the various aspects of the project, especially the preventive therapy aspect, given that this is a new concept to the community members. Support and the collaboration of the Chair of the governing board of the TCV schools, the President of the TCV Schools, and the Minister/Secretary of the CTA Department of Education has been obtained. While conducting the ACF, education regarding TB and Drug Resistant TB shall be provided to the students at the schools. From a past experience from the TB REACH project, we have learned the importance of providing a TB-related education before the start of ACF at the respective school. In addition to educating the students and staff on the modes of TB transmission, diagnosis, treatment, appropriate behaviors, and prevention, we shall incorporate more advanced scientific information as well for students above the 8^th^ grade on the concepts of drug resistance, latent infection, mutations, etc., to generate educational and scientific interest amongst students.

### Active TB Case Finding

The large boarding schools where the students live in congregation provide an ideal opportunity for Active Case Finding of TB. With support from the JHU-CTR, a TB team composed of Delek Hospital TB Medical Officer or a designated Medical Officer, a designated project nurse, and a ACF data manager shall conduct the ACF at the above schools. The TB Medical Officer shall lead the activity at these schools together with the resident medical officer of the TCV schools. The activities shall be carried out in coordination with the administration of the respective schools. For the first year of the project, the students at the TCV schools in the vicinity of Delek Hospital in Dharamsala shall be screened for presence of TB, starting with Lower TCV School in Dharamsala. Into the ACF algorithm is built the mechanism to identify and investigate contacts of existing TB cases. Additionally, contact tracing shall be done to identify contacts of new TB cases at the school. In addition to tracing contacts whenever a new case arise, ACF shall be performed annually in each of the schools. Based upon the case detection rate, ACF may be repeated in a school in the same year. Active Case Finding (ACF) of TB among Tibetan population in India was first initiated in 2011 in collaboration with Johns Hopkins University under the TB REACH grant using a WHO-approved Xpert MTB/Rif Assay (GeneXpert). Xpert MTB/Rif assay is a highly sensitive and specific molecular diagnostic test that can detect Mycobacterium tuberculosis and its resistance to rifampin in clinical specimens in less than two hours. ACF shall be carried out using a pre-defined ACF algorithm (Figure 2), and Xpert MTB/Rif assay shall be employed in testing participants for presence of TB (and rifampin resistance).

**Figure 2**. Diagnostic algorithm for conducting Active TB Case Finding among Tibetan School Children


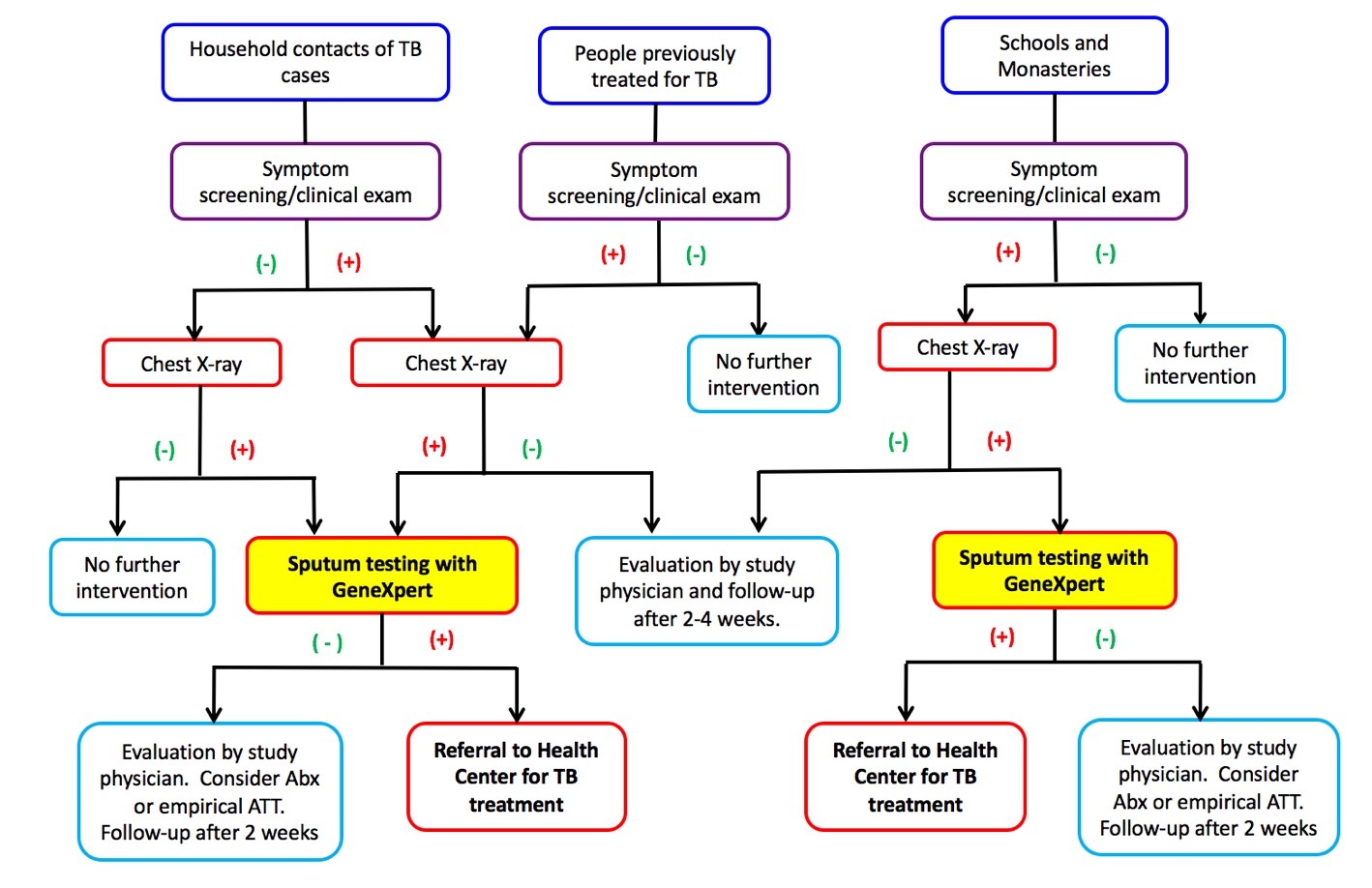


### Contact Investigation

The TB team at Delek Hospital in coordination with the school nurses and health workers shall perform contact tracing of the all the TB cases diagnosed through both the ACF activity and those who routinely present at the hospital. Contacts of TB patients shall be evaluated using a pre-defined algorithm developed for the TB Programme as outlined in the manual of the Tibetan TB Control Programme. A contact investigation register shall be placed at both Delek Hospital and the branch health centers at the school. The contact investigation database and the active case finding database for the students from the participating schools shall be linked through a unique id provided to each student before enrollment into the databases.

### Case Management System

All TB cases detected through the project shall be managed under the setting of the Tibetan TB Control Programme at the Delek Hospital in Dharamsala and Dekyiling Hospital in Uttrakhand for the phase one of the project. Both these hospitals under the CTA-DOH has extensive experience in the management of TB. Delek Hospital particularly has been regularly managing complex TB cases, such as MDR, pre-XDR and XDR-TB cases, some of whom were managed with both bedaquiline and delaminid. Anti-TB treatment is administered under directly observed therapy system. National and international guidelines are followed in treating the drug susceptible and drug resistant TB cases. The MDR-TB cases will be managed in consultation with an Expert TB Panel constituted by members from Delek Hospital, and JHU-CTR.

### Tuberculosis Preventive Therapy

A program for provision of tuberculosis preventive therapy (TPT) for the school children will be developed and implemented. TPT constitutes an important aspect of the model system of TB care. Schoolchildren will be screened for latent TB infection (LTBI) as per the diagnostic algorithm. Tuberculin skin testing will be used to identify latent infection with *M. tuberculosis*, as per the standard of care. The project staff and the health personnel at the schools shall be trained to administer and read the TST. The TB clinic at the Delek Hospital routinely performs TST. After evaluation for active TB disease, latently infected individuals will receive TPT under supervision of the school health nurse. In 2010, the CTA Department of Health has adopted the policy to provide preventive therapy for latently infected individuals with recent exposures to TB, which has been outlined in the manual of the Tibetan TB Control Programme.

- 1. **Project Phases**

### Pre-Implementation

Before the actual implementation of the project, the community shall be educated and sensitized regarding the various aspects of the project, including the preventive therapy for tuberculosis, which is a new concept to the community through the various media and social media platforms.

### Phase I

The Phase I of the project will be carried out in two stages based on geographical proximity of the implementation sites to the Delek Hospital in Dharamsala, where the Project Team will be situated.

- **Stage I**: Implementation in schools nearby Dharamsala (n=7104)
- **Stage II**: Implementation in schools nearby Dekyiling (n=3499)

### Phase II

The Phase II of the project shall be implemented to cover children enrolled in the schools and monasteries outside Himachal Pradesh and Uttrakhand. This phase of the project shall be mostly implemented in the major Tibetan settlements in the state of Karnataka in South India. Mundgod settlement, located in the Karnataka state of India and one of the largest Tibetan settlements, was found to have a very high rate of TB incidence of 486/100,000 during the TB REACH study (2011-2013). In addition to Mundgod, the project shall be implemented to cover children residing in the schools and monasteries in Bylakuppe, Hunsur, and Kollegal Tibetan settlements, from all of which high case rates of TB were detected during the TB REACH project. The total children population will be approximately 4500 for the Project Phase II. The concept, design, and implementation for the Phase II shall remain the same as that of Phase I of the project. The tentative timeline for Project Phase II is from March 2019 through December 2021.

- 1. **Project Population**

The project shall be implemented in the children/students attending the Tibetan schools and monasteries in India. This is a quality improvement project, which builds upon the existing standard of care. For the phase one, the project shall be implemented among students/children studying in the Tibetan residential schools and monasteries in the states of Dharamsala and Uttrakhand. There are 16 schools in these two states with a total student population of approximately 10603. Students in nine schools will be covered in the Stage I of Project Phase I (first year). Students in the remaining six schools will be covered in the Stage II of Project Phase I (second year). Students in the schools are usually under 18 years of age. The staff members in the schools and monasteries who are contacts of TB cases or who have symptoms and signs of TB disease shall also be covered by the project.

- 1. **Endpoints**

### *Primary Outcome Measures*

- TB rates measured over time during active case finding campaigns
- TB preventive therapy initiation and completion rates
  1. **Data Collection, Management, and Confidentiality**

A separate database has been developed for the project to register students screened for LTBI/ TB and treated with preventive therapy or anti-TB treatment. Trained nurses and physicians will conduct in-person interviews and necessary clinical exam, and recommend laboratory test as indicated. A designated project data manager will enter demographic, clinical, and relevant laboratory information of the participants into the Zero TB project database on a daily basis and manage it under the supervision of the project PI at JHU. The data manager will receive prior one-on-one training on entering and management of the database. Values of the variables will be as per the code-book being developed for the project. The data manager shall flag individuals needing follow-up in the project database and ensure timely follow-up of suspected cases. A unique ID number shall be assigned to every participant, which will serve as the unique identifier for the participant. The data shall be anonymized before analysis. Random quality check on the project data will be performed as per the existing CTA DOH quality control guideline for its TB surveillance database. Utmost priority shall be given to maintain confidentiality of the participants.

- 1. **Statistical Analysis Plan**

### Primary outcome variable.

1. Tuberculosis disease
2. Tuberculosis infection

### Secondary outcome variables.

1. Completion rate of TB disease treatment
2. Completion rate of TB Preventive treatment
3. Uptake (Acceptance) of TB preventive treatment
4. Exposure rates to TB cases
   - 1. ***Study Variables and statistical analysis***:

Demographic and social factors include age, sex, institute, settlement/town, district, class, dormitory, birthplace, and year of migration from Tibet. Clinical variables include weight, BCG status, comorbidity, concomitant medications, past TB details, details of TB exposure (time, place, person) at school and home, TB symptoms, and TST reactivity. Laboratory-related variables include details for X-ray, Xpert, culture, and drug susceptibility testing (DST) result. Additional variables related to TPT, TB diagnosis, treatment, and monitoring will be collected. Data are maintained at the Delek Hospital. Descriptive analyses will be performed to characterize TB exposure, infection, and disease. Using age and sex adjusted logistic regressions, relationship between other risk factors and TB infection and disease will be assessed. Time to disease progression will be calculated for children and young adults using survival analysis. Participants are categorized as 1) TST negative (no TBI), 2) TST+ (TBI) receiving TPT, 3) TST+ (TBI) not receiving TPT (refusals and contraindications), and 4) active TB disease. Within this last category, there exists drug-susceptible and drug-resistant TB cases. Schools are 6-monthly and annually screened for TB. Survival analysis will be conducted using Cox Proportional hazard regression for the time-to-event data with time variables served by TPT start- and stop-dates, and TB disease as the outcome. Participants will be censored at the earlier of 1) development of first episode of TB disease, 2) last available follow-up date in the database, or 2) administratively to mark a 5-year follow-up period from March 2017. An interim analysis will be performed after two years of project implementation and as seen appropriate by project team. Hazard ratios will be calculated in participants by comparing 1) TPT vs. no TPT, and 2) TPT with 3HR vs. no TPT, and 3) TPT with 4R vs. no TPT. Incidence trends from 2017-22 will be calculated.

- 1. **Timelin****e**

**Pre-implementation Phase**: September 2016 – February 2017

**Project Phase One**: March 2017 – February 2019 (7104 students)

**Project Phase Two**: March 2019 – February 2021 (3499 students)


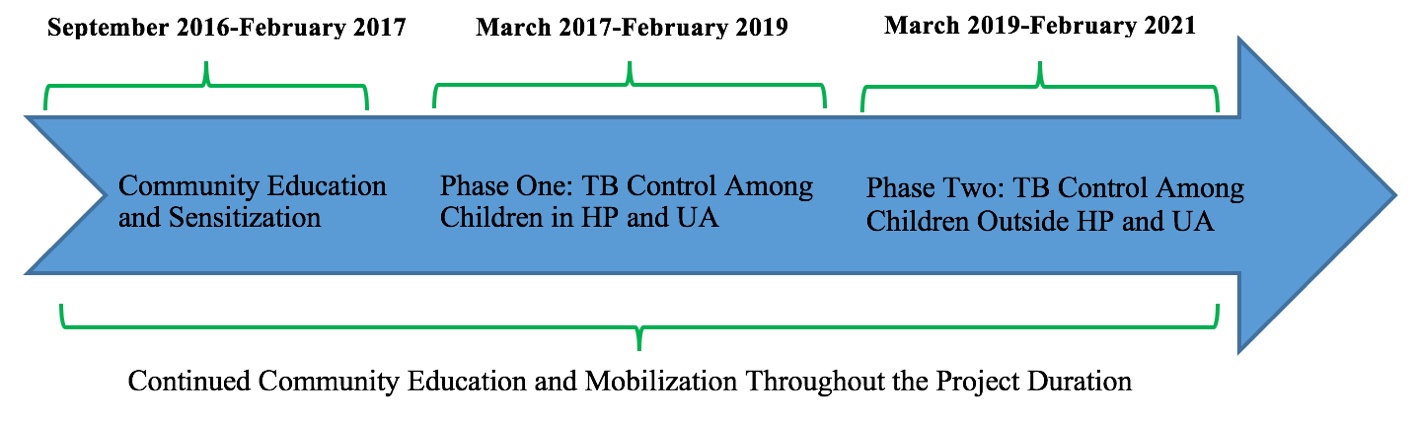


1. **PROJECT RISKS and BENEFITS**

### POTENTIAL RISKS

There are minimal risks associated with this project, as it is being undertaken by the CTA and DOH in partnership with JHU as a way to improve the standard of care.

### KNOWN POTENTIAL BENEFITS

The primary benefits that this project brings to existing standard of care are the technical expertise and resources to what is already available. The project will bring a renewed focus to active TB case finding, and will improve upon surveillance systems and prevention protocols that are already in place.

### RISK/BENEFIT RATIO

Given the minimal risks associated with this study and the potential benefits to society, the benefits outweigh the risks. As for any project, there is a possibility of unknown and unforeseen risk; that possibility is small for this study. If unforeseen risks are recognized during the study, then the sponsor, IRBs/ethics committees, and participants will be provided with relevant information.

## SELECTION AND ENROLLMENT

## *Inclusion Criteria*

Individuals must meet all of the following inclusion criteria in order to be eligible to participate in this quality improvement project:

- All children attending the Tibetan schools.
- Staff members at the schools who are contacts of TB cases or have symptoms/signs of TB.

## *Exclusion Criteria*

Any subjects meeting any of the following exclusion criteria at baseline will be excluded from TB screening activities:

- None

1. **REFERENCES**

1. Nelson L, Naik Y, Tsering K, Cegielski JP. Population-based risk factors for tuberculosis and adverse outcomes among Tibetan refugees in India, 1994–1996. Int J Tuberc Lung Dis. 2005;9(9):1018-26.

2. Salvo F, Dorjee K, Dierberg K, Cronin W, Sadutshang TD, Migliori GB, et al. Survey of tuberculosis drug resistance among Tibetan refugees in India. Int J Tuberc Lung Dis. 2014;18(6):655-62. doi: 10.5588/ijtld.13.0516. PubMed PMID: 24903934.
