# Supplementary figures and images for: Risk of developing active tuberculosis following tuberculosis screening and preventive therapy for Tibetan refugee children and adolescents in India: An impact assessment
Source: PLoS Med. 2021 Jan 19;18(1):e1003502. doi: 10.1371/journal.pmed.1003502 (PMC7853467; doi:10.1371/journal.pmed.1003502)

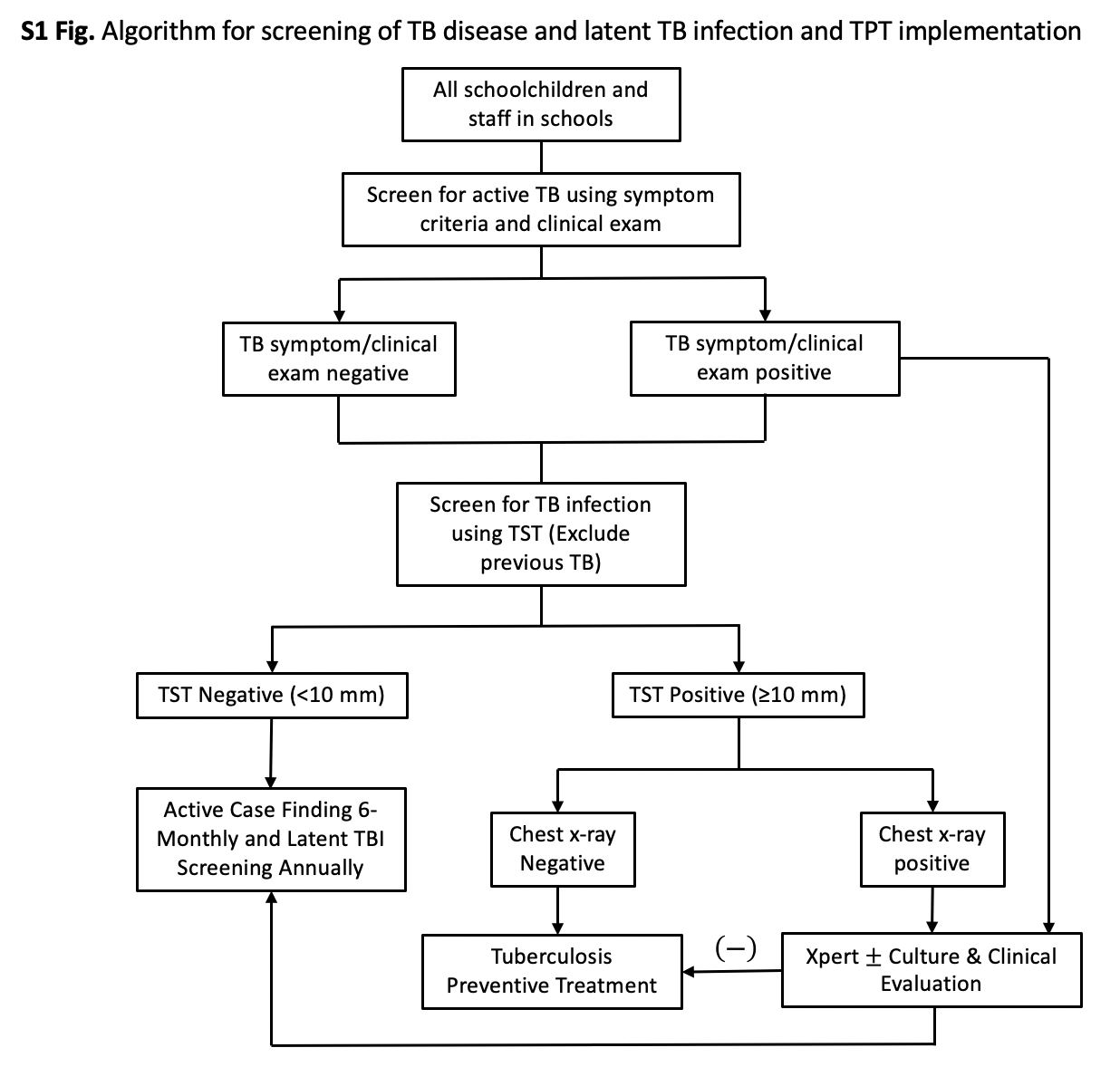

Supplement: S1 Fig — (TIF) [file pmed.1003502.s003.tif]
