# Supplementary material for: Risk of developing active tuberculosis following tuberculosis screening and preventive therapy for Tibetan refugee children and adolescents in India: An impact assessment
Source: PLoS Med. 2021 Jan 19;18(1):e1003502. doi: 10.1371/journal.pmed.1003502 (PMC7853467; doi:10.1371/journal.pmed.1003502)
Supplement: S1 Table — (DOCX) [file pmed.1003502.s004.docx]

**S1 Table. Baseline characteristics of adult staff members that did and did not receive tuberculosis preventive treatment (TPT) in Tibetan boarding schools, Himachal Pradesh, India (2017-2019)**

| **Characteristics (n)** | **All participants**  **(N= 807), n (%)** | **Received TPT (N=125),**  **n (%)** | **Did not receive TPT (N=682), n (%)** | **Chi Square**  **p value** |
| --- | --- | --- | --- | --- |
| Follow up years, median (IQR) | 2.5 (2.3-2.5) | 2.5 (2.3-2.6) | 2.5 (2.3-2.5) |  |
| Total person-years of follow up | 1800 | 240 | 1560 |  |
| Age, median (IQR) | 40 (33-48) | 39 (32-45) | 40 (33-48) | – |
| **Sex**  Female  Male | 467 (57.9)  340 (42.1) | 71 (56.8)  54 (43.2) | 396 (58.1)  286 (41.9) | 0.792 |
| **Calendar year**  2017  2018  2019 | 706 (87.5)  1 (0.12)  100 (12.4) | 89 (90.4)  0 (0.0)  36 (9.6) | 593 (87.0)  1 (0.1)  88 (12.9) | 0.533 |
| **Occupation**  Teacher  Home-mother  Office staff  Other staff | 376 (46.6)  128 (15.9)  94 (11.7)  209 (25.9) | 62 (49.6)  20 (16.0)  9 (7.2)  34 (27.2) | 314 (46.0)  108 (15.8)  85 (12.5)  175 (25.7) | 0.408 |
| **Place of birth**  India  Tibet  Nepal  Bhutan | 539 (66.8)  258 (32.0)  8 (1.0)  2 (0.3) | 89 (71.2)  36 (28.8)  0 (0.0)  0 (0.0) | 450 (66.0)  222 (32.6)  8 (1.17)  2 (0.29) | 0.435 |
| **Weight (kg), median (IQR)** | 65 (57-74) | 65 (57-75) | 65 (58-74) | – |
| **History of previous TB treatment**  Previous TB treatment  Previous MDR TB treatment | 149 (18.5)  2/149 (1.3) | 0 (0.0)  0 (0.0) | 149 (18.5)  2/149 (1.3) | <0.001  0.448 |
| **TB Contact in past 2 years**  TB Contact at school/home  No Contact at school/home | 103 (12.8)  704 (87.2) | 24 (19.2)  101 (80.8) | 79 (11.6)  603 (88.4) | 0.019 |
| **BCG vaccine status**  Received BCG vaccine  Did not receive BCG vaccine | 733 (90.8)  72 (8.9) | 112 (89.6)  13 (10.4) | 621 (91.1)  59 (8.7) | 0.686 |
| **TST status**  TST negative  TST positive | 266/627 (42)  361/627 (58) | 0 (0.0)  125/361 (35) | 266 (0.0)  236/361 (65) | <0.001 |
| **Co-existing medical conditions**  Asthma  Seizure disorder  Chronic Hepatitis B  Hypertension  Diabetes mellitus  Acid-Peptic Disease | 9 (1.1)  1 (0.1)  47 (5.8)  37 (4.6)  7 (0.9)  12 (1.5) | 3 (2.4)  0 (0.0)  5 (4.0)  4 (3.2)  0 (0.0)  4 (3.2) | 6 (0.88)  1 (0.15)  42 (6.2)  33 (4.8)  7 (1.03)  8 (1.17) | 0.137  0.668  0.344  0.421  0.255  0.085 |

^@^History of MDR-TB in last five years. School nurse/parent contacted for confirmation when a child responded positive to contact with drug resistant TB. Pleural TB successfully treated with first-line ATT with no DST result were considered drug susceptible.

^*^BCG vaccine status (left-arm scar) checked only for people born in Tibet. Persons born outside Tibet were assumed to have received BCG at birth under National Immunization Programme.
