# Supplementary material for: Risk of developing active tuberculosis following tuberculosis screening and preventive therapy for Tibetan refugee children and adolescents in India: An impact assessment
Source: PLoS Med. 2021 Jan 19;18(1):e1003502. doi: 10.1371/journal.pmed.1003502 (PMC7853467; doi:10.1371/journal.pmed.1003502)
Supplement: S2 Table — (DOCX) [file pmed.1003502.s005.docx]

**S2 Table. Treatment completion among schoolchildren and staff who were receiving TB preventive therapy using either 3 months of isoniazid and rifampin or 4 months of rifampin.**

| **TPT Regimen** | **TPT Outcome^*^** | **All participants (N=1158), n (%)** | **Children and adolescents (N=1043), n (%)** | **Staff members**  **(N=115), n (%)** |
| --- | --- | --- | --- | --- |
| All TPT | TPT completed  TPT stopped prematurely  Loss to follow | 1138 (98.3)  10 (0.86)  10 (0.86) | 1023 (98.0)  10 (1.0)  10 (1.0) | 115 (100)  0 (0.0)  0 (0.0) |
| 3HR | TPT completed  TPT stopped prematurely  Loss to follow | 834/844 (98.8)  4/844 (0.5)  6/844 (0.7) | 747/757 (98.7)  4/757 (0.5)  6/757 (0.8) | 87/87 (100)  0/87 (0.0)  0/87 (0.0) |
| 4R | TPT completed  TPT stopped prematurely  Loss to follow | 304/314 (96.8)  6 /314 (1.9)  4 /314 (1.3) | 276/286 (96.5)  6/286 (2.1)  4/286 (1.4) | 28/28 (100)  0/28 (0.0)  0/28 (0.0) |

^*^TPT was ongoing for 159 participants, 149 students and 10 staff; 33 on 3HR and 126 on 4R.
