# Supplementary material for: Risk of developing active tuberculosis following tuberculosis screening and preventive therapy for Tibetan refugee children and adolescents in India: An impact assessment
Source: PLoS Med. 2021 Jan 19;18(1):e1003502. doi: 10.1371/journal.pmed.1003502 (PMC7853467; doi:10.1371/journal.pmed.1003502)
Supplement: S3 Table — (DOCX) [file pmed.1003502.s006.docx]

**S3 Table**. Side-effects associated with tuberculosis preventive treatment (TPT) regimens consisting of 3HR (3-months of daily isoniazid and rifampin) or 4R (4-months of daily rifampin) in children and adolescents

| **Side-effect** | **Children (5-14 years)** | | | **Adolescents (10-19 years)** | | |
| --- | --- | --- | --- | --- | --- | --- |
|  | **3-months of isoniazid and rifampin N=376, n (%)** | **4-months of rifampin (4R), n (%)** | **Chi Square**  **p value** | **3-months of isoniazid and rifampin (3HR), n (%)** | **4-months of rifampin (4R), n (%)** | **Chi Square p value** |
| Gastrointestinal^1^ | 16 (4.23) | 0 (0.0) | <0.001 | 47 (6.67) | 0 (0.0) | <0.001 |
| Central Nervous System^2^ | 27 (7.14) | 5 (1.59) | 0.001 | 97 (13.76) | 12 (4.18) | <0.001 |
| Rash/Acne | 7 (1.85) | 0 (0.0) | 0.015 | 24 (3.40) | 0 (0.0) | 0.002 |
| Hepatotoxicity | 0 (0.0) | 1 (0.14) | 0.273 | 2 (0.28) | 1 (0.35) | 0.866 |
| Tiredness | 12 (3.17) | 1 (0.32) | 0.006 | 58 (8.23) | 3 (1.05) | <0.001 |

^1^Gastrointestinal side-effects: nausea, vomiting, gastritis, heartburn, decreased appetite, diarrhea.

^2^Central nervous systems side-effects: headache, dizziness, sleepiness, forgetfulness.
